# Dermoscopy in Female Androgenic Alopecia: Method Standardization and Diagnostic Criteria

**DOI:** 10.4103/0974-7753.58555

**Published:** 2009

**Authors:** Adriana Rakowska, Monika Slowinska, Elzbieta Kowalska-Oledzka, Malgorzata Olszewska, Lidia Rudnicka

**Affiliations:** 1 Department of Dermatology, CSK MSWiA, Woloska 137, Poland; 2 Department of Dermatology, Warsaw Medical University, Koszykowa 82 a, Poland; 3 Division of Health Sciences, Warsaw Medical University, Poland

**Keywords:** Alopecia, dermoscopy, hair, trichoscopy, videodermoscopy

## Abstract

**Objective:**

Establishing the trichoscopy criteria of female androgenic alopecia (FAGA).

**Design:**

Trichoscopy images were retrospectively evaluated.

**Setting:**

Dermatologic hospital-based clinic and private practice offices.

**Patients and methods:**

One hundred and thirty-one females (59 with androgenic alopecia, 33 with chronic telogen effluvium (CTE), 39 healthy controls). The diagnosis was based on clinical examination and confirmed by histopatology.

**Main Outcome Measure:**

Trichoscopy results obtained in frontal, occipital and both temporal areas of the scalp under a 20-fold and 70-fold magnification, including average hair thickness, number of 'yellow dots' and vellus hairs, number of hairs in one pilosebaceous unit and percentage of follicular ostia with perifollicullar hyperpigmentation.

**Results:**

Average hair thickness in frontal area versus occiput was, respectively, 0.061 ± 0.008 mm versus 0.058 ± 0.007 mm in healthy controls, 0.047 ± 0.007 mm versus 0.052 ± 0.008 mm in androgenic alopecia and 0.056 ± 0.007 mm versus 0.053 ± 0.009 mm in CTE. Mean percentage of thin hairs (< 0.03 mm) in androgenic alopecia was 20.9 ± 12% and was significantly higher than in healthy controls (6.15 ± 4.6%, *P* < 0.001) or in CTE (10.4 ± 3.9%, *P* < 0.001). The number of yellow dots, pilosebaceous units with only one hair and with perifollicular hyperpigmentation was significantly increased in androgenic alopecia. Classification and Regression Tree Analysis was performed to establish diagnostic criteria for FAGA.

**Conclusion:**

FAGA may be differentiated from CTE based on trichoscopy criteria. Major criteria are ratio of (1) more than four yellow dots in four images (70-fold magnification) in the frontal area, (2) lower average hair thickness in the frontal area compared to the occiput and (3) more than 10% of thin hairs (below 0.03 mm) in the frontal area. Minor criteria encompass increased frontal to occipital ratio of (1) single-hair pilosebaceous units, (2) vellus hairs and (3) perifollicular discoloration. Fulfillment of two major criteria or one major and two minor criteria allows to diagnose FAGA based on trichoscopy with a 98% specificity.

## INTRODUCTION

Chronic hair loss frequently affects female patients, but there is little or no objective technology available to aid the dermatologist in setting a proper diagnosis and in monitoring treatment efficacy.[1] In particular, it may be difficult to differentiate between female androgenic alopecia (FAGA), a subtype of female pattern hair loss, and chronic telogen effluvium (CTE).[2]

Telogen effluvium is a self-limiting process and almost never causes obvious baldness,[3] whereas FAGA progresses in time, leading to a significant decrease in hair thickness, which over time may become cosmetically unacceptable and psychologically frustrating.[4]

Differences in natural history, prognosis and emerging new therapeutic possibilities[5] make differential diagnosis and early diagnosis of FAGA especially important. Currently, the diagnosis of FAGA is usually based on anamnesis and clinical findings, such as pattern of increased hair thinning, retention of the frontal hairline, and the presence of miniaturized hairs.[6,7] The semi-invasive technique of hair root analysis (trichogram) has a decreasing number of advocates among dermatologists. It is considered to be a poor indicator of FAGA and its severity.[8]

A scalp biopsy examination is usually performed to confirm the diagnosis of FAGA in clinically doubtful cases.[2,3] Histopathology findings in FAGA include a decrease in terminal/vellus hair ratio and decline in mean total follicle count with increasing grade of hair loss. This method also has limitations in everyday practice. Typically, punch biopsies are taken for vertical and horizontal embedding. These are 7-12 mm^2^ in size and contain only a small number of hair follicles. Thus, some authors suggest performing multiple biopsies for representative sampling, which increases the invasiveness of this diagnostic technique and makes it even less useful for monitoring of treatment efficacy.[8]

Trichoscopy[9,10] is a newly developed method of hair image analysis, based on videodermoscopy of the hair and scalp. The method allows visualization of hair shafts at high magnification and performing measurements, such as hair shaft thickness, without the need of removing hair for diagnostic purposes. It also allows *in vivo* visualization of the epidermal portion of hair follicles and perifollicular epidermis.[11,12] Several reports raise the issue of potential usefulness of this technique in diagnosing hair and scalp disorders, such as microsporiasis,[13] androgenic alopecia,[14] alopecia areata,[15,16] lipedematous alopecia,[17] pediculosis[18] or inherited hair shaft abnormalities,[19,20] but the method has not been standardized yet and no criteria for diagnosing acquired diseases of hair and scalp have been established.

### Objective

The aim of the study was to establish a standardized method of trichoscopy in acquired hair loss and to establish trichoscopy criteria for diagnosing FAGA.

### Design

Trichoscopy images from female patients with FAGA, CTE and healthy volunteers collected in the years 2005-2007 were retrospectively evaluated.

### Study selection

The diagnosis was established by clinical examination and histopathology. FAGA was clinically suspected in cases of frontal accentuation ("Christmas tree" pattern), diffuse central or vertex/frontal (male pattern) with sparing of the occiput.[6] The clinical diagnosis of CTE was based on diffuse form of scalp hair thinning longer than 6 months.[3]

All patients suspected for FAGA or CTE had three 4-mm punch biopsy specimens taken from the immediately adjacent skin on the mid scalp and all specimens were sectioned horizontally. The terminal to vellus hair ratio (T:V) at the midisthmus level was used to set the diagnosis. The ratio equal or lower than 4:1 was indicative for FAGA and equal or higher than 8:1 together with anagen: Telogen ratio lower than 8:1 was indicative for CTE.[4]

### Setting

Dermatologic hospital-based clinic and private practice offices.

## PATIENTS AND METHODS

After obtaining approval from the hospital's review board, a total of 131 female patients were included in the study, 59 with FAGA, 33 with CTE and 39 healthy volunteers.

The mean age was 36.2 (18-59) years in patients with FAGA, 32.2 (18-56) years in patients with CTE and 37.8 (19-58) years in healthy controls. The differences were statistically not significant.

Trichoscopy has been performed with the Fotofinder II videodermoscope, which permits scalp visualization at a 20-160-fold magnification. The device is equipped with software that allows to carry out measurements of structures visualized in magnified images and provides results in real scale. Images of the scalp were taken at a 20- fold magnification, which allows high-quality enlargement of 1 cm^2^ of the scalp area to the size of a computer screen and at a 70-fold magnification, which magnifies in a similar manner an area of 9 mm^2^.

In each patient, one image at a 20-fold magnification and four images at a 70-fold magnification were taken in each of the following four areas: Frontal, occipital, right temporal and left temporal.

Hair thickness was measured at a 70-fold magnification, in direct proximity to follicular orifices. Hairs have been identified as 'thin hairs' (below 0.03 mm), 'medium-size hairs' (0.03-0.05 mm) and 'thick hairs' (above 0.05 mm).

The images have been evaluated in accordance with the scheme presented in Table 1.

**Table 1:** Scheme of the trichoscopic images evaluation in the presented study

Parameter	Method of evaluation
Vellus hairs	Number of vellus hairs calculated in one field of vision (FOV) at 20-fold magnification
	Total number of vellus hairs in four FOVs at 70-fold magnification
	Highest result in one FOV from the above (at 70-fold magnification)
Distribution of hair thickness	Percentage of thin hairs (< 0.03 mm)
	Percentage of medium-sized hairs (0.03 − 0.05 mm)
	Percentage of thick hairs (above 0.05 mm)
	Mean hair thickness (mm)
Pilosebaceous units	Percentage of single-hair units (at 20-fold magnification)
	Percentage of double-hairs units
	Percentage of triple-hairs units
Yellow dots	Number of yellow dots per FOV calculated at 20-fold magnification
	Number of yellow dots per FOV calculated in four FOV at 70-fold magnification
	Highest result from the above (at 70-fold magnification)
Perifollicular yellow discoloration (hyperpigmentation)	Percentage of follicular ostia with perifollicular yellow discoloration calculated 20-fold magnification
	Number of follicular ostia with perifollicular yellow discoloration in four FOVs at 70-fold
	Highest result from the above (at 70-fold magnification)
Other	Number of white dots (scarified follicular ostia), cadaverized hairs, broken hairs
	Degree of desquamation (rated 0-4)
	Type of blood vessels
	Presence of exclamation mark hairs

Statistical analysis was performed with the use of Student's *t*-test for paired samples and with analysis of variance (ANOVA). The Classification and Regression Tree technique was used to establish diagnostic criteria.

## RESULTS

### Hair thickness

**Figure 1:**
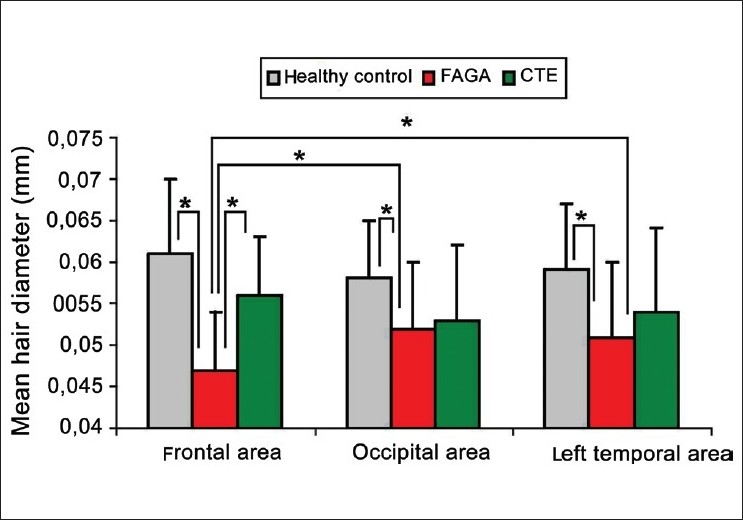
Mean hair diameter in frontal, occipital and left temporal areas of patients with female androgenic alopecia (FAGA), chronic telogen effluvium (CTE) and healthy controls. Asterix indicates the most important, statistically significant differences between them (P < 0.001)

Both in healthy controls as well as in patients with CTE, the thickest hairs have been observed in the frontal area whereas the thinnest in the occipital area. In the healthy control group, the mean hair thickness was 0.061 ± 0.008 mm in the frontal area vs. 0.058 ± 0.007 mm in the occipital area (*P* < 0.001). In telogen effluvium, the values were 0.056 ± 0.007 mm vs. 0.053 ± 0.009 mm, respectively (*P* < 0.001). In FAGA, the smallest average thickness of hair roots has been observed in the frontal area, with 0.047 ± 0.007 mm compared to 0.052 ± 0.008mm in the occipital area (*P* < 0.001) [Figure 1].

Differences between the left and right temporal areas were statistically not significant in any of the investigated parameters. Thus, results for only one (left) temporal area are presented in figures.

In all assessed areas, the smallest average thickness of the hair roots has been noted in patients with FAGA [Figure 1]. Compared with healthy controls, hair thickness of patients with FAGA was significantly reduced in the frontal (*P* < 0.001), occipital (*P* = 0.002), left temporal (*P* < 0.001) and right temporal area (*P* < 0.001).

The largest average percentage of thin hairs (below 0.03 mm) was observed in FAGA in the frontal area (20.9 ± 12%) and it was significantly different compared to patients with telogen effluvium (10.4 ± 3.9%) and healthy volunteers (6.15 ± 4.6%, *P* < 0.001). An increase in the percentage of thin hairs was accompanied by an increase in the proportion of medium-sized hairs and a simultaneous decrease in the proportion of thick hairs. As the process occurred, the shift toward an increase of vellus hairs has also been observed in the occiput of FAGA patients.

**Figure 2:**
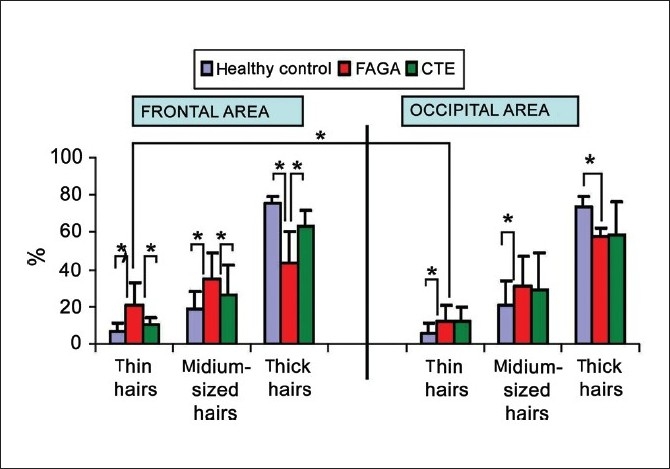
Percentage of thin, medium-sized and thick hairs in frontal and occipital areas in healthy controls, patients with female androgenic alopecia (FAGA) and chronic telogen effluvium (CTE). Asterix marks statistically significant differences (P < 0.05)

The mean proportion of thin, medium-sized and thick hairs in the occipital area of FAGA patients was 14.2 ± 8.9%, 31.5 ± 15.8% and 54 ± 4.9%, respectively. In the control group, the respective proportions were 6 ± 5.1%, 20.6 ± 13.4% and 73.4 ± 5.8%. In telogen effluvium, the respective values were 11.9 ± 3.9%, 29.7 ± 15.9% and 58.6 ± 8.5% [Figure 2].

**Figure 3:**
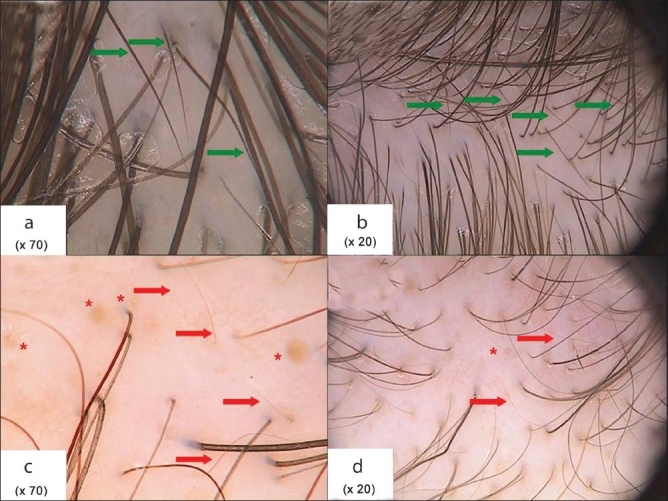
Trichoscopy of frontal scalp area in androgenic alopecia (a and b) and chronic telogen effluvium (c and d). Images presented in 70-fold (a and c) and 20-fold magnification (b and d). Red arrows: Vellus hairs; green arrows: Short, new anagen hairs; red asterix: Yellow dots

Image analysis showed that thin hairs (hairs below 0.03 mm) differ in CTE from FAGA. In CTE thin hairs are short, sharp ended, and become gradually thinner from their proximal to distal end. They are newly regrowing hairs. [Figure 3 and b]. In FAGA thin hairs correspond to vellus hairs resulting from progressive hair follicle miniaturization. They are evenly thin and bluntly ended [Figure 3c and d]. A number of five or more short and sharp-ended hairs in four fields of vision at a 70-fold magnification in both the frontal and the occipital or temporal area was found in 18/33 (54.5%) patients with CTE and in none of the patients with FAGA.

### Pilosebaceous units

Hairs usually are present in groups of few hair roots growing from one follicular orifice. The number of hairs in pilosebaceous units has been evaluated by trichoscopy at the 20-fold magnification. The percentage of single-hair, double-hair and triple-hair units was evaluated.

In healthy controls and in CTE, the highest proportion of single-hair pilosebaceous units was observed in the temporal areas. The mean percentage of single-hair units in this area was 42 ± 12% in CTE and 32 ± 15% in healthy controls.

**Figure 4:**
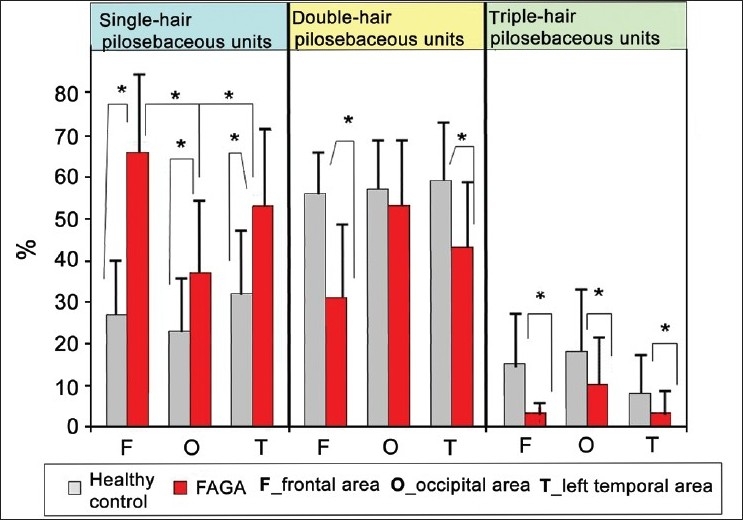
Percentage distribution of pilosebaceous units with one, two and three hairs. In chronic telogen effluvium patients as well as in the healthy controls, the distribution was similar. Thus, only healthy control results and patients with androgenic alopecia are presented

In patients with FAGA, the mean percentage of single-hair pilosebaceous units was highest in the frontal area (65.2 ± 19.9%). This was significantly more than in telogen effluvium (39.0 ± 13.4%, *P* < 0.001) and in healthy controls (27.3 ± 13%, *P* < 0.001). The smallest difference between these groups in the proportion of single-hair pilosebaceous units has been noted in the occipital region. The numbers for healthy controls, FAGA and telogen effluvium were 22.6 ± 12.6%, 36.8 ± 18.6% and 31 ± 23%, respectively [Figure 4] . The difference between FAGA and healthy control was statistically significant at *P* < 0.001. The mean proportion of single-hair units in the frontal area to occipital area was 2.56 in healthy controls, 3.4 in telogen effluvium (*P* = 0.01) and 10.4 in FAGA (*P* < 0.001).

### Yellow dots

**Figure 5:**
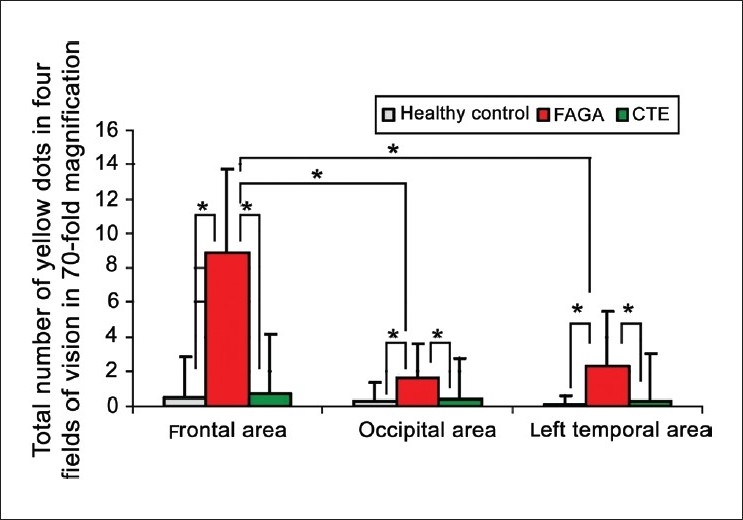
Yellow dots in frontal, occipital and left temporal areas of all three groups of patients, presented as a number counted in four field of visions at 70-fold magnifications. Asterix marks statistically significant differences (*P* < 0.001)

Yellow dots were evaluated at a 20-fold magnification and at a 70-fold magnification. Results are given as total number of yellow dots in one field of vision (FOV) at the 20-fold magnification and as total number seen in four FOVs at the 70-fold magnification. In both calculation methods, the number of yellow dots was significantly higher in patients with FAGA as compared with healthy controls or with patients with CTE. However, in general, the number of yellow dots per 1 mc^2^ was on average 20% higher when evaluated at the 70-fold magnification, as compared with the 20-fold magnification. This difference resulted from better visualization of small trichoscopy structures at higher magnifications. Figure 5 shows the mean numbers of yellow dots in healthy controls, FAGA and telogen effluvium when analyzed in four fields of vision at a 70-fold magnification. The highest number of yellow dots in patients with FAGA was noted in the frontal area (8.86 ± 4.8/4 fields of vision at the 70-fold magnification). The corresponding number in the occipital area was 1.59 ± 2.0.

## PERIFOLLICULAR DISCOLORATION

The percentage of hair-containing units with perifollicular discoloration was evaluated at the 20-fold magnification and in four FOVs at the 70-fold magnification. Both methods yielded highly comparable results. Thus, results are presented for the 20-fold magnification only.

Perifollicular discoloration was found significantly more often in FAGA as compared with healthy controls or patients with CTE. The mean percentage of hair follicles with surrounding discoloration in FAGA was 32.4 ± 4.7% in the frontal area and 6.6 ± 2% in the occipital area (*P* < 0.001).

## OTHER PARAMETERS

Other parameters (white dots, cadaverized hairs, broken hairs, degree of desquamation, predominant type of blood vessels, presence of exclamation mark hairs) were evaluated in accordance with the scheme presented in Table 1 and gave results that were non-significant for setting up criteria for the diagnosis of FAGA.

## CLASIFICATION AND REGRESSION TREE

Data obtained in this study were used to define diagnostic trichoscopy criteria for FAGA by employing a Classification and Regression Tree analysis. These allowed to define three major and three minor criteria into an algorithm, which gave a 98% method specificity for FAGA.

### Major criteria

1. More than four yellow dots in four images at a 70-fold magnification in the frontal area.
2. Lower average hair thickness in the frontal area in comparison with the occiput (calculated from not less than 50 hairs from each area).
3. More the 10% of thin hairs (below 0.03 mm) in the frontal area.

### Minor criteria

1. Ratio of single-hair unit percentage, frontal area to occiput >2:1
2. Ratio of number of vellus hairs, frontal area to occiput >1.5:1
3. Ratio of hair follicles with perifollicular discoloration, frontal area to occiput >3:1.

Fulfillment of two major criteria or one major and two minor criteria is required to diagnose FAGA based on trichoscopy.

In order to confirm this diagnostic model, a linear regression analysis was performed in which the dependent variable was the diagnosis of FAGA and the diagnostic criteria were independent variables. An analytical model, which was constructed in this manner showed a potential diagnostic sensitivity of this method at the level of 72%.

## TRICHOSCOPY REPORT

Based on results collected in this study, we established a trichoscopy report form that contains most important trichoscopy findings in diffuse hair loss and diagnostic criteria for FAGA [Table 2]. Elements, which were not found to be of diagnostic value in this or previous studies,[11,12] were not included in this trichoscopy report form. These include trichoscopy results from parietal regions and results from magnifications that were less precise or significantly less comfortable to handle.

**Table 2:** Trichoscopy report scheme

	Calculations in frontal and occipital areas	FAGA criteria
Major criteria	Total number of yellow dots in four fields of vision*	>4 in frontal area
	Mean hair thickness (mm)*	Lower in frontal area
	Percentage of hairs*	More than 10% thin hairs in the frontal area
	Thin hairs (<0.03 mm)	
	Medium-sized hairs (0.03 − 0.05 mm)	
	Thick hairs (>0.05 mm)	
Minor criteria	Percentage of units in one field of vision**	Ratio of single-hair units, frontal area to occiput > 2:1
	Single-hair unit	
	Two-hair units	
	Three-hair units	
	Total number of vellus hairs in four fields of vision*	
	Percentage of hair follicles with perifollicular discoloration**	Ratio of frontal area to occiput > 1.5:1
	Other observations (i.e., exclamation hairs, cadaverized hairs, white dots)	Ratio of frontal area to occiput > 3:1

* 70-fold magnification,

** 20-fold magnification

## DISCUSSION AND CONCLUSIONS

FAGA, a disease in the spectrum of female-pattern hair loss, is characterized by progressive miniaturization of hair follicles, mediated most probably by dihydrotestosterone within the follicle, and may affect women with normal levels of circulating androgens.[21] The diagnosis is usually based on anamnesis and clinical findings, such as early age of onset, the pattern of increased hair thinning over the frontal/parietal scalp with greater hair density over the occipital scalp, retention of the frontal hairline and the presence of miniaturized hairs.[6,7,22] Histologically, the disease is characterized by miniaturization of a proportion of follicles and an increased percentage of hair in telogen in the affected area.[7] In advanced stages of disease, when these features are obvious, the diagnosis is not problematic. However, in early disease and in patients in whom other causes of hair loss coexist, the diagnosis may be challenging.

In this study, trichoscopy criteria were established, which allow to diagnose FAGA with 98% specificity. These criteria are based on findings that have partly been known from clinical observations and other diagnostic methods but could not be quantified before trichoscopy was developed. These findings relate to predominance of hair miniaturization in the frontal area compared with the occiput.

This clinical and histopathological observation has been identified and quantified by trichoscopy as lower average hair thickness in the frontal area in comparison with the occiput, more the 10% of thin hairs (below 0.03 mm) in the frontal area and ratio of vellus hair number (frontal area to occiput) above 1.5:1.

A novel approach to quantify hair density in practical dermatology is evaluation of the number of hairs in one pilosebaceous unit. This was previously not possible with the classical diagnostic method or the recently developed phototrichogram.[23] The number of hairs in one pilosebaceous unit varies from one to three in healthy persons. Occasionally, a four-hair unit may be found. Our results show that the number of single-hair pilosebaceous units is significantly increased in the frontal area in patients with FAGA compared with the occiput (a ratio above 2:1). Hair thinning, decreased number of hairs in pilosebaceous units and predominant prevalence in the frontal area are main features distinguishing FAGA from most other hair diseases, especially alopecia areata.[11]

Clinical 'patterning' in patients with female-pattern hair loss may be with frontal accentuation ("Christmas tree" pattern), diffuse central or vertex/frontal (male pattern) with sparing of the occiput.[6] Recently, studies have demonstrated that in patients with FAGA, changes observed in the occiput are similar to those in the frontal area, but are less pronounced. Using the phototrichogram method, both Ekmekci[24] and van Neste[1] showed decreased hair density and an increased percentage of thin hair roots (<0.04 mm) in the occipital area of FAGA patients compared with healthy controls. Our results also confirm the occurrence of changes typical for androgenic alopecia in other areas than those considered to be 'androgen-dependent.' We have shown that in the occipital area of patients with FAGA, the average hair diameter is significantly decreased, the percentage of vellus hairs is increased and the number of single-hair pilosebaceous units is higher than in the occipital area of healthy controls. Also, other features of FAGA, such as yellow dots and perifollicular discoloration, were found in the occipital area of patients with FAGA but not in healthy controls. Assessment of these trichoscopy features in temporal areas gave intermediate values between results obtained for the frontal region and the occiput in both FAGA and CTE. It may be concluded that trichoscopy of temporal areas may be ignored in dermatological practice. Interestingly, it has been observed in healthy controls that the temporal areas have the highest number of vellus hairs and single-hair pilosebaceous units, indicating that physiologically these areas have the lowest hair density.

A major trichoscopy criterion of FAGA is the presence of yellow dots, which reflect hair follicle ostia lacking any hairs (empty hair follicles). It may be suggested that yellow dots in FAGA result from the presence of sebaceous lobules, which in histopathology appear large in relation to the miniaturized follicles.[25] We hypothesize that these sebaceous glands are still active after advanced hair follicle miniaturization and produce sebum, which creates intraepidermal sebum lagoons. These sebum lagoons appear as yellow dots in trichoscopy.

Yellow dots have been previously described in alopecia areata[11,12] and have been recently suggested as indicative of alopecia areata incognita (Kligman's telogen effluvium) in patients with diffuse hair loss.[17] Our results show that yellow dots may be characteristic for a wide spectrum of hair diseases and they may also represent a wider than previously anticipated spectrum of histopathological appearances of follicle ostia and infundibula. Our results show that yellow dots are one of the most important trichoscopy features distinguishing FAGA from CTE.

Another important trichoscopy finding is perifollicular discoloration of the skin. This feature, called by some authors as 'hyperpigmentation' or 'peripilar sign' reflects the presence of perifollicular lymphocytic infiltrates in early androgenic alopecia.[26] Our results confirm the presence of perifollicular discoloration in FAGA, although we found that in FAGA the percentage of hair follicles with this abnormality is significantly higher in the frontal area compared with the occiput. According to statistical analysis, a ratio of hair follicles with perifollicular discoloration and frontal area to occiput higher than 3:1 is highly indicative of FAGA.

CTE has no specific trichoscopy features apart from an increased proportion of short, sharp-ended hairs. A number of five or more in four fields of vision at a 70-fold magnification in both the frontal and another (occipital or temporal) was found highly indicative of CTE. Most likely, these are regrowing hairs in early anagen stage and their increased number indicates an accelerated hair cycle, resulting in an intensive replacement of hair roots. However, CTE may rather be a diagnosis made by exclusion of other causes of hair loss, than by direct fulfillment of specific trichoscopy criteria.

In conclusion, the results of our study indicate that FAGA may be differentiated from CTE and the diagnosis of FAGA may be established based solely on trichoscopy criteria.
